# Data-driven Bayesian networks for risk scenario mapping of Falls from height accidents

**DOI:** 10.1371/journal.pone.0334611

**Published:** 2025-10-14

**Authors:** Jue Li, Tengyao Wang

**Affiliations:** School of Transportation, Changsha University of Science & Technology, Changsha, China; Federal University of Pernambuco: Universidade Federal de Pernambuco, BRAZIL

## Abstract

Falls from height (FFH) represent the most frequent type of accident in the building industry, leading to substantial economic losses and posing serious threats to worker safety. While risk analysis plays a vital role in accident prevention, a more comprehensive understanding of risk can significantly contribute to reducing the occurrence of accidents. To better capture the complexity and uncertainty inherent in risk factors, this study adopted the concept of risk scenarios to investigate the underlying mechanisms and driving factors associated with FFH accidents. A total of 368 FFH accident reports from 2014 to 2024 were collected, and a Bayesian network model was developed based on the validated data extracted from these reports. Through this model, various risk scenarios of FFH accidents were systematically explored. The analysis identified five core dimensions influencing the occurrence of FFH accidents, along with five high-risk variables within these dimensions. Moreover, the study examined the probabilities of FFH accidents under different risk scenarios. This scenario-based approach offers new insights into construction safety management and provides valuable implications for enhancing FFH accident prevention strategies in construction projects.

## 1. Introduction

The construction industry is an important part of the global economy. As a labor-intensive sector, it is characterized by complex work environments and frequent interactions among personnel, which can increase overall risk exposure. Among various accidents, Falls from height (FFH) are identified as one of the most frequent accidents, accounting for over 30% of construction accidents [1]. Since working at height is an integral aspect of modern construction practices, FFH accidents have led to significant losses for both society and individual families. They may adversely affect public confidence in safety measures. Consequently, the accurate identification and prevention of FFH accidents remain key concerns in the construction industry [2].

The variability of construction environments and the complexity of interrelated risk factors may limit the effectiveness of current preventive measures [3]. Although technical and managerial approaches have contributed to a reduction in the frequency of accidents overall, the evolving nature of risk determinants still presents challenges. The efficacy of risk prevention strategies was demonstrated to be a significant factor in the reduction of accidents [4]. Zhang et al. [3] critique the predominant focus on isolated risk factors or simple correlations in existing research, noting this approach neglects the dynamic evolution of risk over time. Analyzing the probability of different risk scenarios under varying conditions may offer a more comprehensive understanding of how these risk factors interact [5].

A risk scenario refers to a specific combination of conditions that can negatively affect a system or project, extending beyond the limitations of static risk assessments by capturing nonlinear interactions between risk factors and accident mechanisms. This approach may provide a more complete basis for understanding and managing FFH accidents. However, if risk scenarios are not adequately defined, the overall comprehension of risk may be compromised [6]. To date, few studies have comprehensively examined FFH accident risk scenarios using mathematical modeling, even though the likelihood of such accidents can vary considerably across different scenarios. While expert judgment can inform scenario development [7], objective methods are generally preferred.

To address these gaps, this study considers the following research questions: (1) Which risk variables best represent FFH accidents in construction projects? (2) What are the primary causes of FFH accidents when the interrelationships among risk variables and their associated probabilities are taken into account? (3) Which risk scenarios are associated with a high probability of FFH accidents? The findings of this study are expected to contribute to the existing literature on FFH accident risk analysis and provide practical recommendations for safety managers.

To answer these questions, this study adopts an integrated methodological framework combining Grounded Theory (GT) and Bayesian Networks (BN), supplemented by Dempster-Shafer (D-S) evidence theory and conditional independence testing. GT was employed to systematically extract key risk variables from qualitative accident narratives, leveraging its strength in identifying critical factors in contexts where structured data are limited or fragmented. These GT-derived variables formed the foundation for constructing the BN, which provides a robust framework for quantifying probabilistic relationships among variables and assessing the likelihood of various accident scenarios under uncertainty. The BN structure was developed by identifying potential causal relationships among GT-derived risk variables and validated through a questionnaire survey to ensure accuracy. To mitigate uncertainty and subjectivity in expert judgments, D-S evidence theory was integrated with conditional independence testing, enabling the synthesis of multiple expert opinions while optimizing the BN structure to reflect true conditional dependencies. BN parameters were directly inferred from the accident report dataset, ensuring empirical grounding of the model. This GT–BN–D-S framework was chosen for its ability to address common challenges in accident causation modeling, such as data sparsity, subjective bias, and inaccurate dependency assumptions. Similar approaches have been successfully applied in safety domains like mine water inrush hazards [8] and hazardous materials transportation [9], demonstrating the reliability and adaptability of this methodology for analyzing complex accident systems.

The paper is organized as follows. Section 2 presents a review of the relevant literature. Section 3 explains the research methodology. Section 4 details the results of the study. Section 5 discusses the findings. Section 6 outlines the study’s limitations and suggests avenues for future research, and Section 7 concludes the paper.

## 2. Literature review

In this section, we review research on FFH accident risk in construction projects, examining the main technical approaches, key findings, and current challenges in risk identification, as well as highlighting the methodological limitations and knowledge gaps in the existing body of literature.

Regarding FFH accident risk identification, Shi et al. [10] developed an AHP fuzzy risk evaluation model based on hierarchical analysis and a fuzzy comprehensive evaluation method; their model validation results were consistent with field observations, although their study did not explicitly focus on the specific risk variables associated with FFH accidents. In another work, Nadhim et al.[11] reviewed 297 articles on FFH accidents and, after screening, analyzed 75 articles to identify common risk variables such as individual factors, organizational factors, scaffolding issues, and weather conditions. Similarly, Feng [12] identified 18 risk variables relevant to FFH accidents using safety specifications combined with accident causation theory. By distributing questionnaires to 350 frontline workers and employing SPSS for reliability and validity testing along with confirmatory factor analysis via structural equation modeling, the study found that unsafe worker behavior was the most significant risk factor, followed by the effects of external objects, and thereafter by management and environmental influences.

Alizadeh et al. [13] quantified the posterior probabilities of 37 FFH risk factors by fully embedding expert causal knowledge, a real accident database with a systematic sensitivity test into the same Bayesian network and validated the key drivers of the model through a sensitivity analysis, which showed that non-compliance with work-at-height safety instructions (0.127), lack of safety equipment for work-at-height (0.094) and working at heights work lack of safety instructions (0.071) as significant factors for FFH accidents. This provides a methodological paradigm for causal quantification and critical factor identification of BN in the field of occupational safety. In a similar effort, Zermane et al. [14] identified and quantified risk variables through data collection and fault tree analysis, reporting that failure to wear personal protective equipment (85.93%), inadequate supervision and leadership (89.84%), and non-adherence to or inability to follow work standards (85.15%) were the key risk factors. However, both studies treated the relationships between risk variables as independent and linear, not accounting for their dynamic characteristics. For example, in the context of FFH accidents, risk variables such as A (e.g., illegal construction), B (e.g., inadequate supervision), and C (e.g., careless operation) do not influence accident outcomes in a strictly additive or isolated manner. Depending on whether each variable is present or absent in a given scenario, their interactions may produce very different risk outcomes. This leads to a combinatorial explosion in possible risk states, and the resulting network of influences is inherently dynamic and non-linear.

Peng et al. [7] employed Bayesian network models in conjunction with gray-scale decision laboratory and explanatory modeling techniques to identify factor relationships, concluding that low safety awareness is the most critical risk factor in FFH accidents. Their results further suggest that the interactions among risk variables are dynamic and non-linear rather than independent and linear. More recently, Qi et al. [15] constructed a BN structural model based on the mature HFACS (Human Factors Analysis and Classification System) framework in the aviation industry by integrating multi-source AI technology to achieve automatic causal extraction and structure learning, and correcting the algorithmic output with expert knowledge. The subjectivity and inefficiency of traditional manual extraction are solved. Finally, in the BN node quantification, the probability of each node is jointly quantified by data statistics and EM algorithms, and the sensitivity analysis further screens the key nodes, which in turn leads to the high-fall and high-risk factors in the workplace, and promotes the transformation of BN from a theoretical model to an engineered automated tool. Extensive literature—including Zhang et al.‘s factor identification framework [16] and Lee & Kim’s predictive modeling [17]—has centered on identifying potential risk variables and developing risk identification methods. Few studies have examined the dynamic relationships among risk variables or quantified risk probabilities under various risk scenarios. Evaluating risk scenarios with different probabilities can reveal the dynamic interactions between risk factors, offering a more comprehensive view of how FFH accident risks evolve over time. Such an approach provides a theoretical basis for decision-making and improved accident prevention, ultimately contributing to the safety of construction personnel. For example, some studies have explored the automatic prediction and prevention of high-risk FFH accidents using computer vision [18,19] and real-time monitoring through wearable belt devices and BIM data [20]. While these techniques rely on graphical semantics and real-time worker localization to activate preventive measures as established in the field’s methodological conventions, risk scenarios capture the dynamic interplay between risk variables in greater detail and offer a more complete understanding of the conditions underpinning prevention strategies.

In conclusion, our study introduces a novel data-driven hybrid approach to FFH accident risk analysis, combining grounded theory with BN to propose a data-driven “risk scenario mapping” framework. Specific innovations include: (1) node identification: applying the grounded theory to accident reports, extracting risk variables directly from FFH accident reports and generating a frequency table of risk variables; (2) relationship establishment: designing a questionnaire based on accident reports and historical documents, covering multiple subjects such as project managers, construction administrators, associate researchers, site managers, and frontline construction personnel, and introducing evidence theory and conditional independence test to optimize the structure and ensure subjective and objective coordination (3) Probability quantification: relying on the frequency table generated by the rooted theory to directly calculate the node probability. Based on this, the study systematically derives the risk scenarios and high-risk factors of FFH accidents.

## 3. Methods

**Fig 1:**
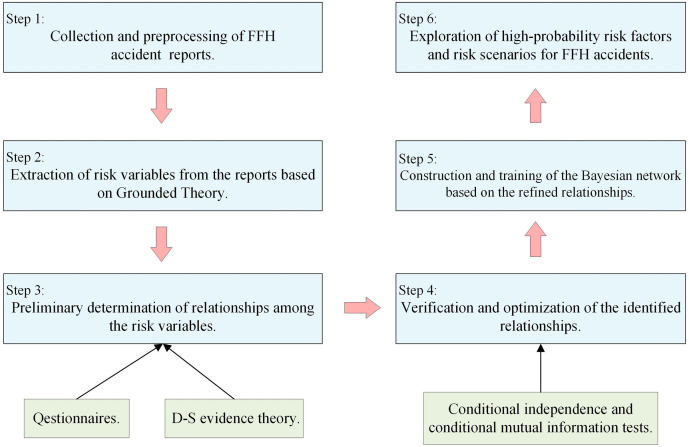
Research framework of the study.

This study was conducted to investigate the risk scenarios associated with FFH accidents in construction projects. A total of 368 FFH accident reports, issued by relevant authorities in China over the last decade, were analyzed for this purpose. The analysis in this study was conducted through the following six sequential steps:(1) Collection and preprocessing of FFH accident reports to ensure data completeness and consistency;(2) Extraction of risk variables from the reports based on Grounded Theory;(3) Preliminary determination of relationships among the risk variables through expert questionnaires and integration using Dempster–Shafer evidence theory;(4) Verification and optimization of the identified relationships by applying conditional independence and conditional mutual information tests with objectively reported accident data to reduce subjectivity;(5) Construction and training of the Bayesian network based on the refined relationships [21], with parameters calculated from the occurrence frequencies of risk factors identified in the GT analysis; and(6) Exploration of high-probability risk factors and risk scenarios for FFH accidents through probabilistic inference. The detailed research framework, illustrating these six sequential steps within a data-driven approach, is presented in Fig 1.

### 3.1. Grounded theory

**Fig 2:**
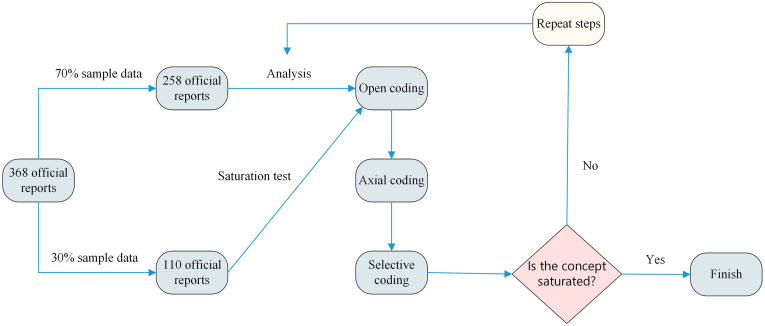
The detailed steps of the Grounded Theory.

This study employed Grounded Theory to identify risk factors from FFH accident reports. Grounded Theory was originally proposed by Glaser and Strauss (1967) in a study on handling terminally ill patients in hospitals [22]. It provides a systematic process of data collection and analysis to help construct theory from actual data [23], allowing new insights without being limited by existing frameworks. Over time, various methodologies have emerged within Grounded Theory; we mainly compare the two approaches of the founders [24], namely that of Glaser and that of Strauss. Glaser’s approach emphasizes the natural discovery of theories from data and is better suited for exploratory research. In contrast, Strauss and Corbin expanded on this by providing a more detailed, step-by-step framework that allows for a clearer extraction of risk variables from FFH accident reports. For our purposes, we adopted the Strauss and Corbin approach. Their method consists of open coding, axial coding, and selective coding [25]. We also conducted a saturation test [26] to ensure comprehensive risk factor identification. The detailed steps used in this study are presented in Fig 2.

**Fig 3:**
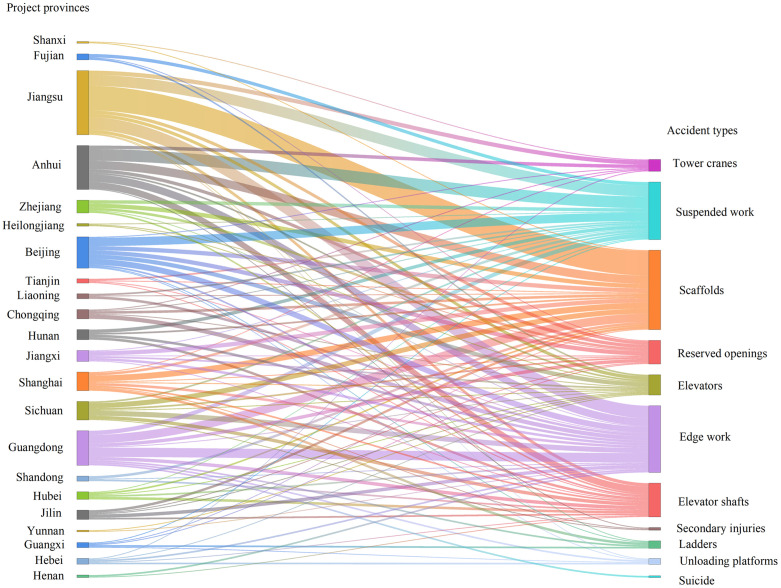
Detailed distribution of the 368 reports.

In the study, we collected 368 FFH accident reports issued by China’s construction-related authorities over the last decade. These reports, written by expert investigation teams and released by government agencies, contain detailed accounts of the specific projects, times, locations, processes, and casualties involved in each FFH accident. Given their official nature, these reports follow standardized documentation procedures and offer valuable domain-specific narratives suitable for in-depth qualitative analysis. However, it should be noted that all data were sourced from a single national context—namely, China. As such, the style, focus, and terminology in the reports may reflect region-specific regulatory practices and administrative priorities. This raises the possibility of documentation bias and limits the immediate generalizability of our findings to other countries or construction cultures. The reports cover 23 provinces (e.g., Beijing, Shanghai, Hunan, Guangdong, Jiangxi, Anhui) and include various types of projects such as commercial buildings, industrial plants, subways, exterior renovations, and interior renovations. Our selection criteria were as follows: (1) the accident must have occurred in the last 10 years for timeliness; (2) the project should be representative, meaning the contract amount exceeds 10 million yuan; (3) the accident report must be published by the relevant government agency through formal channels. We also categorized the types of FFH accidents, with details presented in Fig 3.

**Fig 4:**
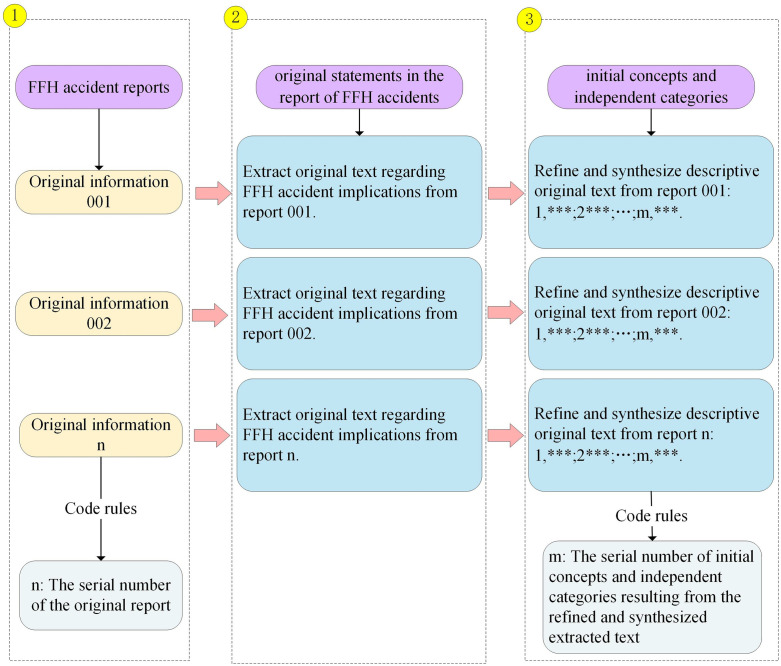
The detailed steps of open coding.

Open coding is an analytical process for developing initial concepts and independent categories from the data [27]. The collected accident reports were first arranged in chronological order, with the earliest report labeled 001 and the most recent one labeled 368. A line-by-line reading was then conducted for each report, beginning with 001, to extract all original expressions relevant to fall-from-height (FFH) accidents. This extraction process was carried out sequentially until all reports had been reviewed. Following the initial extraction, the researcher returned to report 001 to refine and integrate the extracted expressions, aiming to generate more precise and concise initial concepts and independent categories. This refinement process was also conducted in numerical order and continued until the entire dataset was processed. To improve the clarity and transparency of this procedure, Fig 4 is provided to illustrate the full workflow of coding, concept development, and category formation.

**Fig 5:**
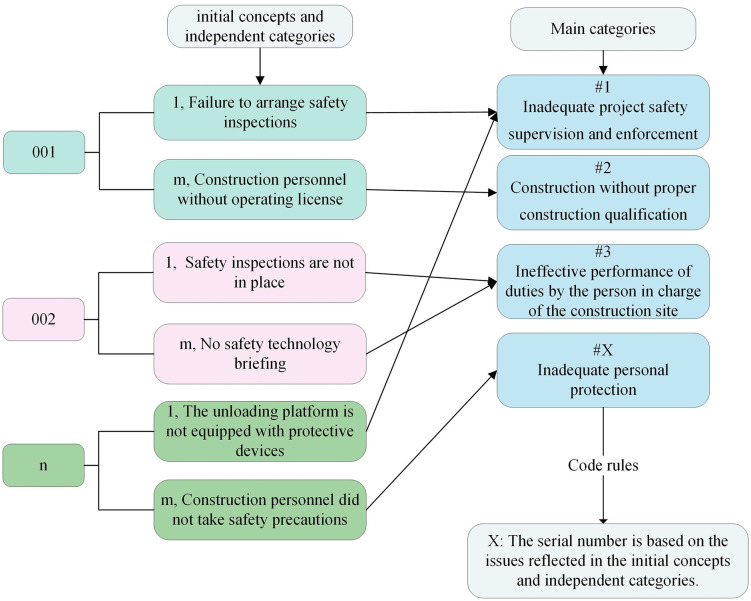
The detailed steps of axial coding.

Axial coding involves grouping similar codes from open coding to identify relationships among them and form higher-level concepts [28]. Based on the initial concepts and independent categories refined during the open coding phase, further integration and categorization were conducted in the axial coding stage to develop more precise main categories. To illustrate this process clearly, an example is presented in Fig 5. In this example, the initial concepts and independent categories derived from reports 001, 002, and n during open coding are assumed to be 1 and m, respectively. The axial coding process proceeds sequentially from report 001 to report n: (1) The process begins with report 001, where its initial concepts and independent categories are integrated and grouped to form preliminary main categories (e.g., Main Category #1 and Main Category #2); (2) Report 002 is then analyzed in comparison with the existing main categories. If its initial concepts and categories align with any existing main category, they are assigned accordingly. If not, a new main category (e.g., Main Category #3) is established; (3) This process continues in report numbering order. For each subsequent report, its concepts and categories are either integrated into the existing main categories or used to create new ones, depending on their similarity and relevance.

Through this iterative process, a complete set of main categories is developed. These main categories are then marked under each corresponding report. For instance, as shown in Fig 2, the results of this categorization are as follows: Report 001: Main Category #1, #2; Report 002: Main Category #3; Report n: Main Category #1, #X.

Selective coding is similar to that of axial coding but involves a higher level of integration, comparison, and refinement. The researcher systematically examined all previously developed main categories to identify and construct core categories. These core categories serve as the central organizing themes that integrate and support the main categories, which in turn are grounded in the more basic initial concepts and independent categories. Through this hierarchical structure, a coherent theoretical system is established, providing a clearer and more accurate understanding of the underlying risk mechanisms in FFH accidents.

A saturation test was performed to ensure that all relevant concepts were identified and to decide when to stop data analysis. The 368 reports were divided into two sets: 258 preliminary samples and 110 saturation test samples. After open, axial, and selective coding were completed on the preliminary sample, the saturation sample underwent the same process to verify that no new significant categories or concepts emerged [5]. If no new categories were found, the theory was considered saturated; otherwise, the analysis would be repeated until saturation was achieved.

### 3.2. Constructing risk variable relationships for FFH accidents

There are multiple methods for constructing relationships between risk variables in FFH accidents, such as Bayesian networks [7], frequency analysis, the analytic hierarchy process [29], latent class analysis [30], logistic regression analysis [31], correlation analysis, and text mining techniques. In this study, we use a Bayesian network, and the relationships among risk variables were constructed within this framework.

#### 3.2.1. Methods for building risk variables based on Bayesian networks.

For Bayesian networks, there are three main ways to establish relationships between risk variables: (1) constructing their causal relationships based on expert knowledge [30]; (2) automatic learning based on sample data to construct causal relationships [32]; (3) a hybrid approach that combines expert knowledge to provide a prior structure or constraints, and then optimizes or validates the Bayesian network with sample data [33]. This study adopts the last approach, which combines expert knowledge and data optimization. This approach not only makes full use of expert knowledge from practical experience but also makes full use of objective factual data, which well balances the relationship between subjectivity and objectivity and provides sufficient and reliable preparation for the subsequent construction of Bayesian networks.

#### 3.2.2. Preliminary establishment of risk factor relationships using expert knowledge.

1) Questionnaire

To establish risk factor relationships, the accident reports were analyzed in detail. Based on this analysis, a series of questionnaires was developed. The respondents who filled out the questionnaires were divided into two groups. One group, labeled Expert Teams, consisted of industry specialists with more than 10 years of experience working on construction projects, including project managers, directors of construction administration, research associate professors, and construction site managers. The other group, labeled Frontline Teams, consisted of industry practitioners with more than 2 years of experience working on construction projects, including project safety officers, construction workers, and other frontline project staff.

A total of 11 questionnaires were distributed to the Expert Team and 45 to the Frontline Team. In these questionnaires, respondents are asked about the influence relationships between variables on a scale of 0–10, where 0 indicates that there is no relationship between factor X and factor Y, and 10 indicates that factor X must cause factor Y to occur. Finally, respondents were also allowed to suggest additional relationships that they believe exist but were not listed.

2) D-S evidence theory

D-S evidence theory, formally known as Dempster-Shafer evidence theory, is a mathematical tool for dealing with uncertainty and is used as a technique for synthesizing information from different sources to arrive at a reasonable and objective view of an observed object [34]. We mainly use D-S evidence theory to effectively integrate the questionnaire information from two different sources, expert teams and frontline teams, to improve the accuracy of risk relationships. Dempster’s rule of combination is the D-S theory’s method for fusing different sources of information. The fusion formula for different information sources is as follows:


$$m(A)=\frac{\text{1}}{1-k}\sum {\mathrm{\backslash nolimits}}_{X\cap Y=A,X\cap Y\neq \text{\textbackslash{}O}}m_{1}(X)m_{2}(Y)\;\;\;\;\;\;\;\;\;\;\;\;\;\;\;\;\;\;\;\;\;\;\;\;k=\sum {\mathrm{\backslash nolimits}}_{X\cap Y=\text{\textbackslash{}O}}m_{1}(X)m_{2}(Y)$$ (1)

Where m_1_(X) and m_2_(Y) denote the subsets X and Y of support for source 1 and source 2, respectively,m(A) denotes the value of support for the same question from a mixture of different sources.k is the conflict coefficient, which represents the degree of conflict between the two sources of evidence. Next, we calculated the Belief Function, denoted Bel, which represents the level of confidence in the hypothesis; and the Plausibility Function, denoted Pl, which represents the maximum possible level of confidence in hypothesis A given the evidence. These coefficients can help this study to synthesize different sources of information and select reliable risk relationships while minimizing subjectivity [35].

#### 3.2.3 Eliminating subjectivity and optimizing risk variable relationships.

1) Conditional independence

**Fig 6:**
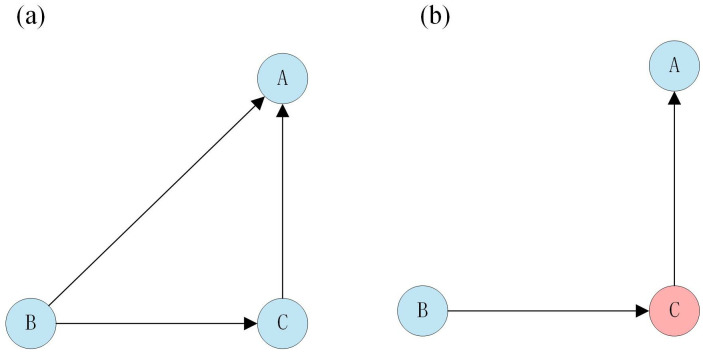
(a) The relationship among variables A, B, and C. (b) Variables A and B are conditionally independent of variable C.

Conditional independence refers to the independence of two variables given a specific condition, even if there may be underlying relationships between them. When the conditioning variable is known, the relationship between the two variables is effectively “blocked,” meaning they no longer directly influence each other. In Bayesian Networks, this assumption allows the joint probability distribution to be factorized into smaller, local conditional probability tables, thereby simplifying computation and facilitating causal interpretation. In this study, conditional independence relationships among variables are represented in Fig 6, where the absence of an edge between two nodes indicates that they are conditionally independent given their respective parent nodes. Previous research has shown that neglecting conditional independence when determining causal links—such as in hazardous material transportation accident modeling—can reduce structural reliability and introduce bias [9]. Because expert judgment obtained through questionnaire surveys may be affected by personal experience, cognitive bias, or recall errors, this study integrated expert assessments with objectively reported accident investigation data. Leveraging the conditional independence property during BN structure helped mitigate the influence of such subjectivity, thereby improving the robustness and credibility of causal link identification. Therefore, to ensure the reliability of the risk variable relationship, the relationship between the risk variables should be modified with the actual data.

Where (a) the relationship among variables A, B, and C is shown, where“→” represents the influence relationship, B → A indicates that variable B causes variable A to occur; and (b) the conditional independence of variables A and B is shown, indicating that the link between variables A and B disappears under the condition of the third variable C, because all of variable B’s influences on variable A are through variable C, which in turn influences variable A.

2) Conditional mutual information

To further verify conditional independence, conditional mutual information was calculated using the frequency of each risk variable in the accident reports. A frequency table was constructed to summarize the main categories, representing all risk variables for FFH accidents, derived through the Grounded Theory process. These main categories, labeled A1 to E3 for clarity, indicate the presence (1) or absence (0) of each risk variable in individual accident reports. Table 1 displays their frequency distribution across the analyzed reports. D-separation was then applied to identify and remove spurious relationships, ensuring that the final network captures only the objective dependencies among variables.

**Table 1:** Frequency statistics for the risk factors in each report.

ID	A1	A2	A3	B1	B2	B3	B4	B5	B6	B7	B8	C1	C2	C3	D1	D2	D3	E1	E2	E3
001	1	0	1	1	1	0	0	0	0	1	0	0	0	1	1	0	0	0	1	0
002	0	0	1	1	1	0	0	1	0	1	0	0	0	1	0	0	0	0	0	0
003	1	0	0	0	1	0	1	0	1	1	0	0	0	0	1	0	1	1	0	0
004	1	1	0	1	1	0	0	0	0	1	0	0	0	0	1	0	1	1	0	0
005	0	0	1	0	1	0	1	0	0	0	0	0	0	0	1	0	0	1	1	0
006	0	0	1	1	1	0	0	0	0	0	0	0	0	0	0	0	0	1	1	0
007	1	1	1	0	0	1	0	0	0	1	0	0	0	0	1	0	1	1	0	0
008	0	0	1	0	1	0	1	0	0	0	0	0	0	0	1	0	1	0	0	0
009	1	1	0	0	1	1	0	0	0	0	0	0	0	0	1	0	1	1	0	0
010	1	1	0	0	1	1	0	0	0	0	0	1	0	1	0	0	1	1	1	1
011	1	1	0	0	1	0	0	0	1	0	0	1	0	1	1	0	0	0	0	0
...																				
364	0	0	1	1	1	0	0	0	0	0	0	0	0	0	0	0	0	0	1	0
365	0	0	0	1	0	0	1	1	0	0	0	0	0	0	0	0	0	1	1	0
366	0	0	0	0	1	0	0	0	0	0	0	0	0	0	1	0	1	0	1	0
367	0	0	0	0	0	0	0	0	0	0	0	0	0	1	0	1	0	1	1	0
368	0	0	0	0	1	0	0	1	0	0	0	0	0	0	0	0	1	0	0	0


$$I(X;Y\;|\;Z)=\sum_{x\in X}\sum_{y\in Y}\sum_{z\in Z}P(x,y,z)\log\frac{P(x,y\;|\;z)}{P(x\;|\;z)P(y\;|\;z)}\;\;\;\;\;\;\;\;P(x,y,z)=\frac{N(x,y,z)}{N}$$ (2)

Where P(x,y,z) is the joint frequency probability distribution of variables X, Y, and Z. P(x,y/z), P(x/z), and P(y/z) are the probabilities of the marginal frequencies. Where N is the total number of samples and N(x,y,z) is the number of joint occurrences of variables X, Y, and Z in the frequency table of a single accident, the probability of the other marginal frequencies is calculated in the same way.

### 3.3. Bayesian network

In this step, the main objective of this study is to explore the risk scenarios of FFH accidents, which requires knowing the relationships between risk variables and risk probabilities. There are many methods to analyze risk relationships and probabilities, such as the causal diagram method, fault tree analysis [36,37], and event tree analysis. The aim of this study is to reflect the complex relationships and dynamics among risk factors of FFH accidents and to reveal the global probabilistic effects caused by changes in risk variables. While the above-mentioned risk analysis methods, except for Bayesian network, focus more on static relationships and are difficult to capture the dynamic change characteristics of risk factors.

In addition to these static methods, other advanced modeling techniques, such as Random Forests, Decision Trees, and fuzzy logic-based approaches, have demonstrated extensive applications in construction safety risk analysis [38–40]. For example, Random Forests have been used for accident type prediction [38], Decision Trees for identifying mortality-related factors in FFH accidents [39], and fuzzy logic-based decision support systems for ergonomic risk assessment [40]. However, these methods primarily focus on classification or rule-based inference, and while they can identify important predictors or approximate decision boundaries, they lack an inherent probabilistic graphical structure to represent conditional dependencies among variables. As a result, they are less suitable for modeling how changes in one factor propagate through a network of interrelated risks.

**Fig 7:**
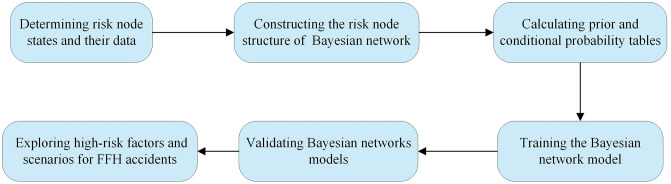
Detailed steps for constructing a Bayesian model for FFH accidents.

Bayesian networks, also known as belief networks, form a directed acyclic graph (DAG) through directed line segments and nodes [41]. The advantage of Bayesian networks over other methods is that the dynamic probability of risk variables can be analyzed through prior probability and conditional probability tables (CPTs) derived from domain knowledge and data, and when a change in one risk variable occurs, the Bayesian network can quickly calculate the impact on the occurrence of other risk variables [42,43]. Another advantage of Bayesian networks is that risk can be visualized through graphical node relationships and probabilistic numerical representations. On this basis, the Bayesian network was selected as the most appropriate approach for this study, as it not only supports the dynamic and probabilistic nature of FFH accident risk analysis but also enables transparent integration of expert knowledge and empirical evidence. The detailed steps of the Bayesian network are shown in Fig 7.

#### 3.3.1. Methods for Bayesian network nodes and their data.

The risk variables identified through Grounded Theory were used as nodes in the Bayesian network. Each node was assigned two states, TRUE and FALSE, indicating the presence or absence of the risk factor. The training data for the Bayesian network model were organized in a frequency table based on these states across all accident reports.

#### 3.3.2. Building the structure of Bayesian network models for risk variables.

Based on the relationships established using expert knowledge and optimized with data-driven methods, the network structure was constructed according to Bayesian network requirements, directly using the risk variable relationships identified in previous steps.

#### 3.3.3. Methods for calculating prior and conditional probabilities of risk variables.

The prior probability and conditional probability are the datasets for training the Bayesian network model [44]. Based on the frequency tables of risk variables of 368 FFH accident reports, and combining the risk variables and different combinations of states of the risk variables, the a prior probabilities and conditional probabilities can be calculated. The detailed steps are as follows: (1) Analyze the structural relationship and different states of the target risk variables; (2) Calculate the risk probabilities of different risk states based on the total number of accident reports, 368, and combined with the frequency table of risk variables. The specific calculation rules are as follows:


$$P(A=TRUE)=\frac{X_{A}+\alpha }{368+2\alpha }$$ (3)

$$P(A=TRUE/C)=\frac{X_{A/C}+\alpha }{N_{C}+2\alpha }$$ (4)

where α is Laplace smoothing, adding this parameter can avoid the probability of 0 to improve the generalization ability of the model; P(A = TRUE) represents the a prior probability when the state of the A node is TURE, X_A_ represents the number of samples when the state of the A node is TURE; P(A = TRUE│C) represents the conditional probability when the A node state is TURE under the combination of the states of the parent node C, X_A_│_C_ is the number of samples when the A node state is TURE under the combination of the states of the parent node C, and N_C_ is the total number of samples for the combination of the states of the parent node C.when some parent node combinations do not appear in the data, the marginal probability of the parent node can be used for estimation, and the geometric mean can be used to calculate the combination probability.

#### 3.3.4. Training of Bayesian network models.

A relational structure was established to represent the Bayesian network. The derived probabilities were manually entered into Netica 5.12, with each node corresponding to a risk variable and linked to a probability table. For root nodes, the entered values represented prior probabilities; for child nodes, conditional probabilities were defined based on frequency-based joint distributions. Once entered, Netica performed probabilistic inference using the belief propagation algorithm. This process updates the posterior probabilities of all nodes according to the input evidence and the specified conditional dependencies, enabling the network to capture complex interrelationships among risk factors. This transparent setup ensures that the model’s quantitative behavior is entirely driven by empirically grounded input values, rather than hidden software assumptions. The resulting network supports scenario analysis, sensitivity testing, and probabilistic reasoning based on both structured expert judgment and observed data patterns.

#### 3.3.5. Validation of Bayesian network models.

The trained Bayesian network model was validated using Netica 5.12 by adjusting the probability values of the risk factors. Three recent FFH accidents from the past six months were selected, and their risk factors were extracted according to the methodology. The model’s predictions were then compared to the actual outcomes to assess its validity.

#### 3.3.6. Exploring the main causes and risk scenarios of FFH accidents.

Bayesian networks allow for bidirectional inference, enabling forward reasoning (predicting accident occurrence) and backward reasoning (identifying accident causes). A diagnostic analysis was performed in Netica 5.12 to calculate the influence probability of each risk factor, thus identifying the most impactful causes of FFH accidents. Additionally, forward inference was applied to analyze the risk probabilities under different scenarios. All risk scenarios were compiled, and those with high accident probabilities were identified. This two-way inference mechanism combines prior knowledge with actual data, dynamically updating probability distributions to reflect complex relationships and risk dynamics.

### 3.4. Ethics statement

This study involved an online questionnaire survey of adult professionals and analysis of publicly available accident reports. No minors were involved. Participants were informed of the study’s purpose, anonymity, and voluntary nature through a welcome page (see Supporting Information, SI_Consent_Form.pdf), where submitting the questionnaire by clicking the ‘开始作答’ button was considered informed consent. Per relevant institutional policies, formal ethics committee approval was not required for anonymized surveys and secondary analysis of public data, ensuring compliance with ethical standards and data privacy regulations.

## 4. Results

### 4.1. Risk variables for Fall from height accidents

#### 4.1.1. Results of open coding analysis of FFH accidents.

**Fig 8:**
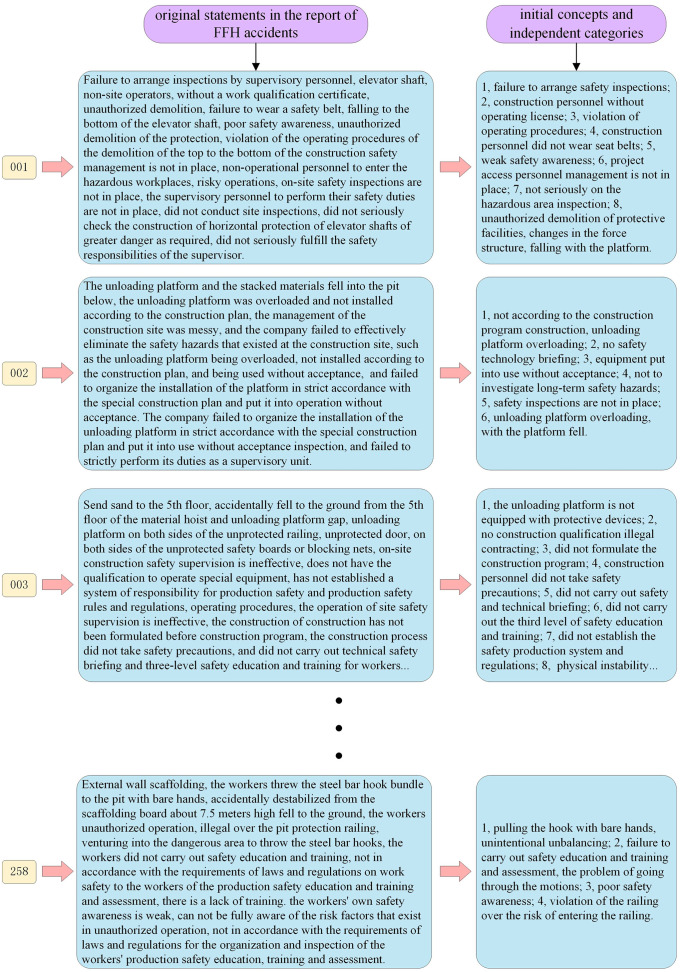
The detailed process of open coding in 258 preliminary samples.

Through the analysis and comparison of tagged statements in the accident reports, risk concepts and associated categories were identified. Fig 8 presents a detailed overview of the open coding process applied to the preliminary 258 reports, as confirmed by the subsequent saturation test. In Fig 8, each yellow box denotes an individual accident report number (e.g., “001” represents the first report that was coded), while the blue boxes display the original statements that were identified and tagged within that specific report. The final blue box in the sequence encapsulates the risk concepts and categories that were distilled through a systematic process of analytical generalization of the tagged statements. This comprehensive visualization helps to illustrate how raw data from the reports was transformed into structured risk factors, thereby laying the foundation for further analysis.

#### 4.1.2 Results of axial coding analysis of FFH accidents.

Axial coding facilitates the transition from descriptive to theoretical coding, allowing the integration of risk information by identifying relationships between categories and contextual information to express risk in more precise elements. This process yielded 20 main categories, including construction without proper construction qualification (A1), ineffective performance of duties by the person in charge of the construction site (B1), and non-standard scaffolding (C1), among others. Detailed information is displayed in the main categories in Table 2.

**Table 2:** Analysis results for selective coding and axial coding.

Core categories	ID	Main categories
(A) Illegal and unauthorized construction practices	A1	Construction without proper construction qualification
	A2	Illegal and unauthorized construction
	A3	In violation of instructions, unauthorized construction
(B) Lack of effective safety management and supervision	B1	Ineffective performance of duties by the person in charge of the construction site
	B2	Inadequate project safety supervision and enforcement
	B3	Inadequate supervision by administrative departments
	B4	Inadequate safety system or unclear responsibility
	B5	Communication and coordination problems
	B6	Technical and design defects or deficiencies
	B7	Inadequate elimination of safety hazards
	B8	Inadequate response to emergencies
(C) Hazardous site environment and equipment	C1	Non-standard scaffolding
	C2	Inadequate site lighting
	C3	Defective work equipment and facilities
(D) Inadequate site security and personal protection	D1	Inadequate personal protection
	D2	Failure to post safety warnings and signs
	D3	Inadequate site safety protection
(E) Risks to the occupational safety of construction workers	E1	Careless operation
	E2	Low safety awareness of construction personnel
	E3	Physical or psychological problems of construction personnel

#### 4.1.3. Results of selective coding analysis of FFH accidents.

Through comparison and analysis of the main categories, five core categories were refined. These core dimensions represent the central risk elements contributing to FFH accidents and offer deeper insight into underlying risk factors. The core categories are listed in Table 2.

#### 4.1.4. Results of saturation test related to FFH accidents.

The 368 accident reports were divided into 258 preliminary samples and 110 samples for saturation testing. After applying open, axial, and selective coding to the preliminary samples, the same coding procedures were repeated on the 110 saturation samples. No new initial concepts or categories emerged from the saturation test, thereby confirming that theoretical saturation was achieved. This indicates that the coding process comprehensively captured the relevant risk factors and that the study passed the theory saturation test.

### 4.2. Result of risk variable relationships for FFH accidents

#### 4.2.1. Results of preliminary determination of risk factor relationships.

The 20 main categories identified through axial coding were defined as risk variables. To establish the relationships among them, 12 and 45 questionnaires were distributed to expert and frontline teams, respectively. A total of 11 and 41 valid responses were received, with response rates of 90.91% and 91.11%. Dempster–Shafer (D-S) evidence theory was used to analyze the data. The belief function (Bel), plausibility function (Pl), and conflict coefficient (k) were calculated, as shown in Table 3.

**Table 3:** Results of the D-S evidence theory.

Number	Risk variables’ relationship	Expert Teams	Frontline Teams	Bel	Pl	k
1	B4 → B2	0.88	0.790	0.9650	0.9650	0.2796
2	B2 → B1	0.84	0.807	0.9564	0.9564	0.2912
3	B2 → C3	0.84	0.754	0.9415	0.9415	0.3273
4	B2 → B7	0.91	0.832	0.9804	0.9804	0.2278
6	B1 → D1	0.81	0.795	0.9430	0.9430	0.3171
7	B1 → C3	0.68	0.771	0.8774	0.8774	0.4024
8	B7 → C3	0.77	0.812	0.9353	0.9353	0.3315
9	B7 → D3	0.77	0.802	0.9313	0.9313	0.3369
10	B7 → D2	0.73	0.768	0.8995	0.8995	0.3767
11	D3 → C3	0.77	0.756	0.9121	0.9121	0.3618
12	D2 → E2	0.67	0.727	0.8439	0.8439	0.4228
13	B6 → C1	0.65	0.761	0.8554	0.8554	0.4217
15	B3 → A1	0.85	0.778	0.9521	0.9521	0.3054
16	A1 → A3	0.88	0.761	0.9589	0.9589	0.3016
17	B8 → B5	0.76	0.737	0.8987	0.8987	0.3768
18	B5 → E1	0.79	0.705	0.8999	0.8999	0.3811
19	A3 → E1	0.90	0.761	0.9663	0.9663	0.2912
20	C2 → E1	0.77	0.717	0.8945	0.8945	0.3828
21	D1 → FFHA	0.84	0.798	0.9540	0.9540	0.2974
22	C1 → FFHA	0.88	0.761	0.9589	0.9589	0.3016
23	C3 → FFHA	0.82	0.751	0.9322	0.9322	0.3394
24	E2 → FFHA	0.84	0.766	0.9450	0.9450	0.3191
25	E1 → FFHA	0.81	0.793	0.9423	0.9423	0.3183
26	E3 → FFHA	0.77	0.744	0.9068	0.9068	0.3682
27	A2 → FFHA	0.84	0.785	0.9504	0.9504	0.3062

**Fig 9:**
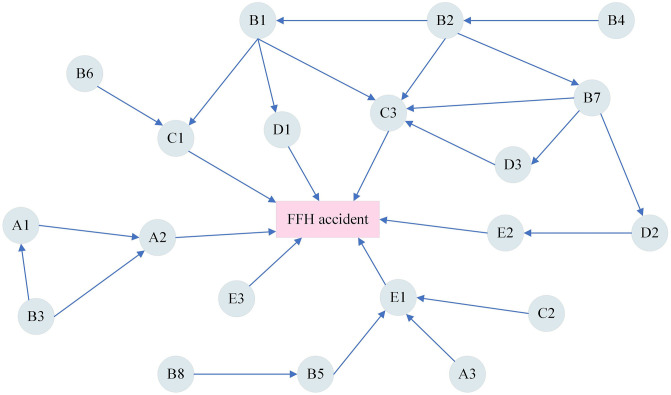
Preliminary determination of risk factor relationships.

In D-S evidence theory, the belief function (Bel) indicates the degree of shared support for different sources of information. The plausibility function (Pl) indicates the maximum degree of support under the condition of no contradiction. And in the conflict coefficient, 0 indicates complete agreement, 1 indicates complete conflict, and a value between 0 and 0.5 is considered to be in the normal range of no conflict between information sources. In Table 3, both the belief function (Bel) and the plausibility function (Pl) are greater than 0.8, and the conflict coefficient k is between 0 and 0.5. Based on the information of D-S evidence theory, the risk factor relationship of FFH accidents is constructed. The resulting risk variable relationships are shown in Fig 9.

#### 4.2.2. Results of eliminating subjectivity and optimizing risk-variable relationships.

As conditional independence is difficult to identify subjectively, the initially established risk relationships were further optimized using D-separation to improve objectivity. Based on Fig 9, conditional independence was tested for specific pairs such as B3 → A2 given A1, and B2 → C3 given B1 or B7. Conditional mutual information was calculated and is shown in Table 4. When the calculated conditional mutual information is less than the threshold value of 0.02 [9], the risk variables are considered to be independent of each other, and vice versa. In Table 4, only the conditional mutual information of B3 → A2 in A1 is larger than 0.02, and the rest are smaller than 0.02. As a result, links such as B2 → C3 and B7 → C3 were removed.

**Table 4:** Results of Conditional mutual information calculations.

Variable impact relationships	Conditional variable	Conditional mutual information
B3 → A2	A1	0.0301
B2 → C3	B1	0.0002
B2 → C3	B7	0.0006
B7 → C3	D3	0.0152

It’s worth mentioning that the initial set of risk factors and their potential interrelationships was first derived from accident narratives through Grounded Theory analysis, ensuring empirical relevance based on actual case data. These relationships were then further evaluated using structured questionnaires involving two respondent groups—domain experts and frontline construction personnel—to capture domain knowledge and practical perspectives. To reduce possible biases introduced by subjective judgment, Dempster–Shafer evidence theory was applied to assess consistency between responses, followed by conditional independence tests to optimize the final BN structure. While the influence of subjectivity in expert-derived relationships cannot be entirely ruled out, the resulting network structure integrates both data-informed insights and practical knowledge. This combination is expected to improve the interpretability and contextual applicability of the model in FFH accident risk assessment.

### 4.3. Result of the Bayesian network model

#### 4.3.1. Results for Bayesian network nodes and their data.

The 20 main categories were used as nodes in the Bayesian network. Each node was assigned two states (TRUE or FALSE), with “FFHA” representing the occurrence of an FFH accident. The frequency table developed earlier served as the data source for conditional mutual information, prior probabilities, and model training

#### 4.3.2. The structure of the Bayesian network model.

The relationship between the risk factors of FFH accidents in the previous subsection was constructed based on the Bayesian network model, and after optimizing the relationships in Fig 9, we constructed this model using Netica 5.12 software.

#### 4.3.3. Results for Prior and Conditional Probabilities of Risk Variables.

**Fig 10:**
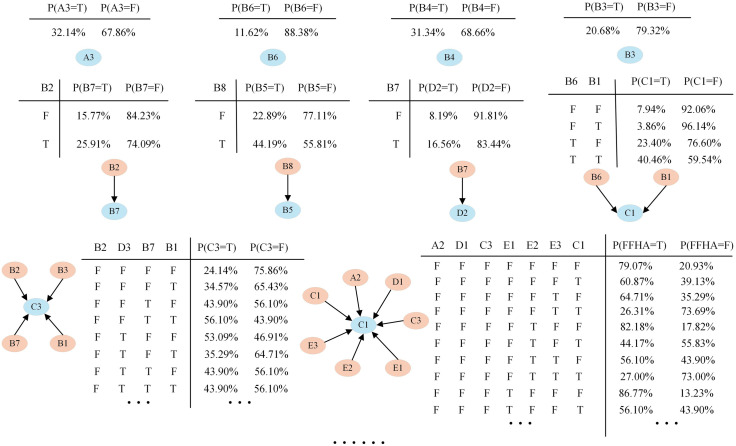
Prior and conditional probabilities of risk variables.

Prior and conditional probabilities were calculated from the frequency table compiled from the 368 accident reports. The resulting probabilities are shown in Fig 10.

#### 4.3.4. Results of Bayesian network model training.

**Fig 11:**
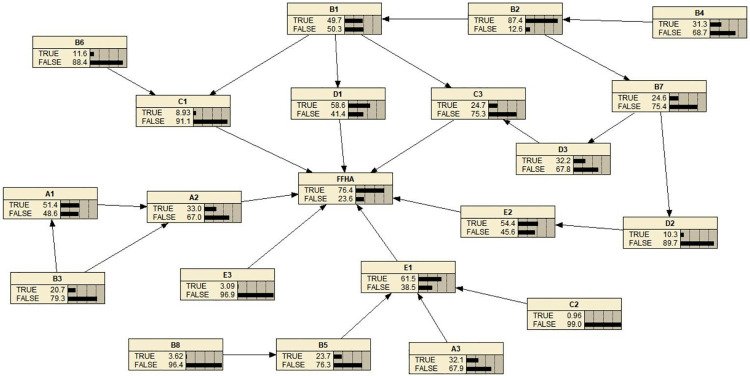
Results of completed Bayesian network model training.

The Bayesian network model was trained using the processed data. The resulting probability distributions of each risk variable are shown in Fig 11. The top five variables with the highest risk probabilities were B2, E1, D1, E2, and A1.

#### 4.3.5. Results of Bayesian network model validation.

**Fig 12:**
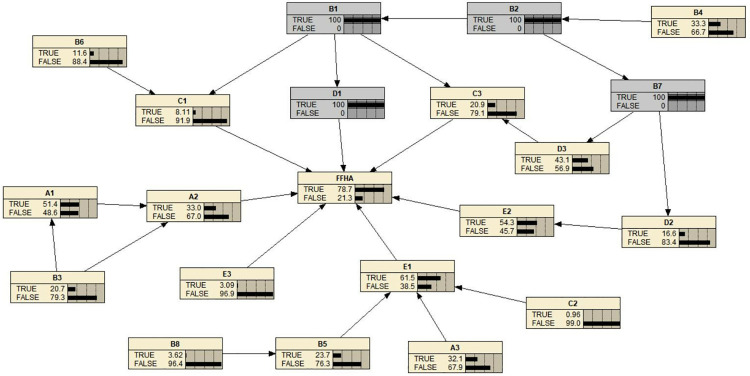
T01 reports predictive results in the Bayesian network model.

We re-searched the official reports of the three FFH accidents that occurred in the last six months, numbered the test reports T01, T02, and T03. Risk variables were extracted following the same grounded theory procedure. The risk variables included in T01 were identified D1, B2, B7, B1; the risk variables included in T02 were A1, A3, B5, D1, D3, B1, B3, A2; and the risk variables included in T03 were found to be E2, D1, B2, B7, E1. By adjusting the probability of the risk variables in the model, the predicted probabilities were 78.7%, 72.2%, and 80.6%, respectively. The prediction details of T01 in the model are presented in Fig 12; T02 and T03 were validated similarly. These prediction accuracies suggest that the model performs reliably.

#### 4.3.6. Results of exploring main causes and risk scenarios with high risk probabilities of FFH accidents.

**Fig 13:**
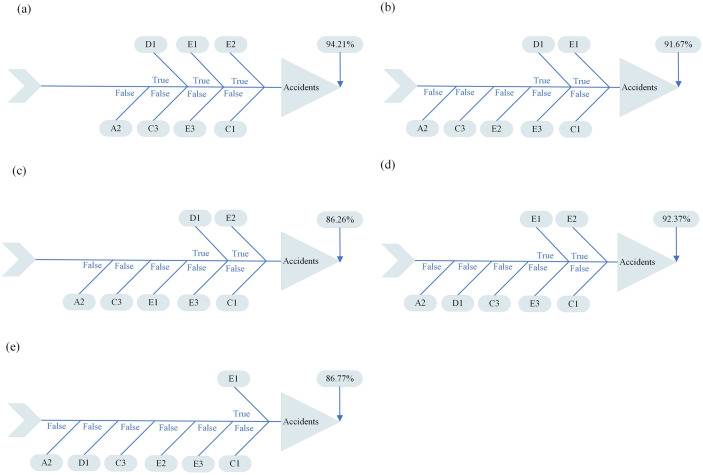
Five high-risk scenarios in this study.

Based on the Bayesian model, risk variables with higher probabilities are more influential in FFH accident occurrence. As shown in Fig 11, the top contributors are B2, E1, D1, E2, and A1. Subsequently, this study predicted the risk scenarios with different probabilities of risk of FFH accidents, and a total of 128 different risk scenarios are shown in Table 5. The five highest-risk scenarios, presented in Fig 13, are summarized. The probability of the first highest risk FFH accident scenario is 94.21% as shown in part a of Fig 13, D1, E1, and E2 are True, A2, C3, E3, and C1 are False. The probability of the second-highest risk scenario is 92.37%, as shown in part d of Fig 13. E1 and E2 are True, A2, D1, C3, E3, and C1 are False. The probability of the third highest risk scenario is 91.67% as shown in part b of Fig 13. D1 and E1 are True, A2, C3, E2, E3, and C1 are False. The probability of the fourth highest risk scenario is 86.77%, as shown in part e of Fig 13. E1 is True, A2, D1, C3, E2, E3, and C1 are False. The fifth highest risk scenario probability is 86.26% as shown in part e of Fig 13, D1 and E2 are True, A2, C3, E1, E3, and C1 are False. These results highlight critical combinations of variables that lead to elevated FFH accident risk, providing meaningful input for targeted prevention strategies.

**Table 5:** Results of various risk scenarios with FFH accidents.

Number	Risk variable states							Probability
	A2	D1	C3	E1	E2	E3	C1	FFHA
1	TRUE	TRUE	TRUE	TRUE	TRUE	TRUE	TRUE	22.16%
2	TRUE	TRUE	TRUE	TRUE	TRUE	TRUE	FALSE	32.01%
3	TRUE	TRUE	TRUE	TRUE	TRUE	FALSE	TRUE	60.87%
4	TRUE	TRUE	TRUE	TRUE	TRUE	FALSE	FALSE	56.10%
5	TRUE	TRUE	TRUE	TRUE	FALSE	TRUE	TRUE	21.59%
6	TRUE	TRUE	TRUE	TRUE	FALSE	TRUE	FALSE	31.19%
7	TRUE	TRUE	TRUE	TRUE	FALSE	FALSE	TRUE	56.10%
8	TRUE	TRUE	TRUE	TRUE	FALSE	FALSE	FALSE	56.10%
9	TRUE	TRUE	TRUE	FALSE	TRUE	TRUE	TRUE	20.57%
10	TRUE	TRUE	TRUE	FALSE	TRUE	TRUE	FALSE	29.70%
11	TRUE	TRUE	TRUE	FALSE	TRUE	FALSE	TRUE	33.64%
12	TRUE	TRUE	TRUE	FALSE	TRUE	FALSE	FALSE	60.87%
13	TRUE	TRUE	TRUE	FALSE	FALSE	TRUE	TRUE	20.04%
14	TRUE	TRUE	TRUE	FALSE	FALSE	TRUE	FALSE	28.94%
15	TRUE	TRUE	TRUE	FALSE	FALSE	FALSE	TRUE	60.87%
16	TRUE	TRUE	TRUE	FALSE	FALSE	FALSE	FALSE	64.71%
17	TRUE	TRUE	FALSE	TRUE	TRUE	TRUE	TRUE	26.26%
18	TRUE	TRUE	FALSE	TRUE	TRUE	TRUE	FALSE	37.93%
19	TRUE	TRUE	FALSE	TRUE	TRUE	FALSE	TRUE	42.96%
20	TRUE	TRUE	FALSE	TRUE	TRUE	FALSE	FALSE	82.18%
21	TRUE	TRUE	FALSE	TRUE	FALSE	TRUE	TRUE	25.59%
22	TRUE	TRUE	FALSE	TRUE	FALSE	TRUE	FALSE	36.96%
23	TRUE	TRUE	FALSE	TRUE	FALSE	FALSE	TRUE	56.10%
24	TRUE	TRUE	FALSE	TRUE	FALSE	FALSE	FALSE	85.71%
25	TRUE	TRUE	FALSE	FALSE	TRUE	TRUE	TRUE	24.37%
26	TRUE	TRUE	FALSE	FALSE	TRUE	TRUE	FALSE	35.20%
27	TRUE	TRUE	FALSE	FALSE	TRUE	FALSE	TRUE	39.87%
28	TRUE	TRUE	FALSE	FALSE	TRUE	FALSE	FALSE	70.49%
29	TRUE	TRUE	FALSE	FALSE	FALSE	TRUE	TRUE	23.75%
30	TRUE	TRUE	FALSE	FALSE	FALSE	TRUE	FALSE	34.30%
		
31	TRUE	TRUE	FALSE	FALSE	FALSE	FALSE	TRUE	38.85%
32	TRUE	TRUE	FALSE	FALSE	FALSE	FALSE	FALSE	74.65%
33	TRUE	FALSE	TRUE	TRUE	TRUE	TRUE	TRUE	56.10%
34	TRUE	FALSE	TRUE	TRUE	TRUE	TRUE	FALSE	30.42%
35	TRUE	FALSE	TRUE	TRUE	TRUE	FALSE	TRUE	60.87%
36	TRUE	FALSE	TRUE	TRUE	TRUE	FALSE	FALSE	56.10%
37	TRUE	FALSE	TRUE	TRUE	FALSE	TRUE	TRUE	20.52%
38	TRUE	FALSE	TRUE	TRUE	FALSE	TRUE	FALSE	29.64%
39	TRUE	FALSE	TRUE	TRUE	FALSE	FALSE	TRUE	33.57%
40	TRUE	FALSE	TRUE	TRUE	FALSE	FALSE	FALSE	60.87%
41	TRUE	FALSE	TRUE	FALSE	TRUE	TRUE	TRUE	19.55%
42	TRUE	FALSE	TRUE	FALSE	TRUE	TRUE	FALSE	28.23%
43	TRUE	FALSE	TRUE	FALSE	TRUE	FALSE	TRUE	31.98%
44	TRUE	FALSE	TRUE	FALSE	TRUE	FALSE	FALSE	56.10%
45	TRUE	FALSE	TRUE	FALSE	FALSE	TRUE	TRUE	19.05%
46	TRUE	FALSE	TRUE	FALSE	FALSE	TRUE	FALSE	27.51%
47	TRUE	FALSE	TRUE	FALSE	FALSE	FALSE	TRUE	56.10%
48	TRUE	FALSE	TRUE	FALSE	FALSE	FALSE	FALSE	70.49%
49	TRUE	FALSE	FALSE	TRUE	TRUE	TRUE	TRUE	24.96%
50	TRUE	FALSE	FALSE	TRUE	TRUE	TRUE	FALSE	36.05%
51	TRUE	FALSE	FALSE	TRUE	TRUE	FALSE	TRUE	40.84%
52	TRUE	FALSE	FALSE	TRUE	TRUE	FALSE	FALSE	76.32%
53	TRUE	FALSE	FALSE	TRUE	FALSE	TRUE	TRUE	24.32%
54	TRUE	FALSE	FALSE	TRUE	FALSE	TRUE	FALSE	35.13%
55	TRUE	FALSE	FALSE	TRUE	FALSE	FALSE	TRUE	39.79%
56	TRUE	FALSE	FALSE	TRUE	FALSE	FALSE	FALSE	70.49%
57	TRUE	FALSE	FALSE	FALSE	TRUE	TRUE	TRUE	23.16%
58	TRUE	FALSE	FALSE	FALSE	TRUE	TRUE	FALSE	33.46%
59	TRUE	FALSE	FALSE	FALSE	TRUE	FALSE	TRUE	37.89%
60	TRUE	FALSE	FALSE	FALSE	TRUE	FALSE	FALSE	64.71%
61	TRUE	FALSE	FALSE	FALSE	FALSE	TRUE	TRUE	22.57%
62	TRUE	FALSE	FALSE	FALSE	FALSE	TRUE	FALSE	32.60%
63	TRUE	FALSE	FALSE	FALSE	FALSE	FALSE	TRUE	56.10%
64	TRUE	FALSE	FALSE	FALSE	FALSE	FALSE	FALSE	72.73%
65	FALSE	TRUE	TRUE	TRUE	TRUE	TRUE	TRUE	25.83%
66	FALSE	TRUE	TRUE	TRUE	TRUE	TRUE	FALSE	37.31%
67	FALSE	TRUE	TRUE	TRUE	TRUE	FALSE	TRUE	56.10%
68	FALSE	TRUE	TRUE	TRUE	TRUE	FALSE	FALSE	72.73%
69	FALSE	TRUE	TRUE	TRUE	FALSE	TRUE	TRUE	25.17%
70	FALSE	TRUE	TRUE	TRUE	FALSE	TRUE	FALSE	36.35%
71	FALSE	TRUE	TRUE	TRUE	FALSE	FALSE	TRUE	41.18%
72	FALSE	TRUE	TRUE	TRUE	FALSE	FALSE	FALSE	64.71%
73	FALSE	TRUE	TRUE	FALSE	TRUE	TRUE	TRUE	23.97%
74	FALSE	TRUE	TRUE	FALSE	TRUE	TRUE	FALSE	34.62%
75	FALSE	TRUE	TRUE	FALSE	TRUE	FALSE	TRUE	64.71%
76	FALSE	TRUE	TRUE	FALSE	TRUE	FALSE	FALSE	79.07%
77	FALSE	TRUE	TRUE	FALSE	FALSE	TRUE	TRUE	23.36%
78	FALSE	TRUE	TRUE	FALSE	FALSE	TRUE	FALSE	33.73%
79	FALSE	TRUE	TRUE	FALSE	FALSE	FALSE	TRUE	60.87%
80	FALSE	TRUE	TRUE	FALSE	FALSE	FALSE	FALSE	79.07%
81	FALSE	TRUE	FALSE	TRUE	TRUE	TRUE	TRUE	30.62%
82	FALSE	TRUE	FALSE	TRUE	TRUE	TRUE	FALSE	60.87%
83	FALSE	TRUE	FALSE	TRUE	TRUE	FALSE	TRUE	50.08%
**84**	FALSE	TRUE	FALSE	TRUE	TRUE	FALSE	FALSE	**94.21%**
85	FALSE	TRUE	FALSE	TRUE	FALSE	TRUE	TRUE	29.83%
86	FALSE	TRUE	FALSE	TRUE	FALSE	TRUE	FALSE	43.08%
87	FALSE	TRUE	FALSE	TRUE	FALSE	FALSE	TRUE	48.80%
**88**	FALSE	TRUE	FALSE	TRUE	FALSE	FALSE	FALSE	**91.67%**
89	FALSE	TRUE	FALSE	FALSE	TRUE	TRUE	TRUE	28.41%
90	FALSE	TRUE	FALSE	FALSE	TRUE	TRUE	FALSE	41.03%
91	FALSE	TRUE	FALSE	FALSE	TRUE	FALSE	TRUE	46.47%
**92**	FALSE	TRUE	FALSE	FALSE	TRUE	FALSE	FALSE	**86.26%**
93	FALSE	TRUE	FALSE	FALSE	FALSE	TRUE	TRUE	27.68%
94	FALSE	TRUE	FALSE	FALSE	FALSE	TRUE	FALSE	39.98%
95	FALSE	TRUE	FALSE	FALSE	FALSE	FALSE	TRUE	45.28%
96	FALSE	TRUE	FALSE	FALSE	FALSE	FALSE	FALSE	83.02%
97	FALSE	FALSE	TRUE	TRUE	TRUE	TRUE	TRUE	24.55%
98	FALSE	FALSE	TRUE	TRUE	TRUE	TRUE	FALSE	35.46%
99	FALSE	FALSE	TRUE	TRUE	TRUE	FALSE	TRUE	40.17%
100	FALSE	FALSE	TRUE	TRUE	TRUE	FALSE	FALSE	72.73%
101	FALSE	FALSE	TRUE	TRUE	FALSE	TRUE	TRUE	23.93%
102	FALSE	FALSE	TRUE	TRUE	FALSE	TRUE	FALSE	34.55%
103	FALSE	FALSE	TRUE	TRUE	FALSE	FALSE	TRUE	56.10%
104	FALSE	FALSE	TRUE	TRUE	FALSE	FALSE	FALSE	67.86%
105	FALSE	FALSE	TRUE	FALSE	TRUE	TRUE	TRUE	22.79%
106	FALSE	FALSE	TRUE	FALSE	TRUE	TRUE	FALSE	32.91%
107	FALSE	FALSE	TRUE	FALSE	TRUE	FALSE	TRUE	56.10%
108	FALSE	FALSE	TRUE	FALSE	TRUE	FALSE	FALSE	67.86%
109	FALSE	FALSE	TRUE	FALSE	FALSE	TRUE	TRUE	22.20%
110	FALSE	FALSE	TRUE	FALSE	FALSE	TRUE	FALSE	32.06%
111	FALSE	FALSE	TRUE	FALSE	FALSE	FALSE	TRUE	56.10%
112	FALSE	FALSE	TRUE	FALSE	FALSE	FALSE	FALSE	74.65%
113	FALSE	FALSE	FALSE	TRUE	TRUE	TRUE	TRUE	29.10%
114	FALSE	FALSE	FALSE	TRUE	TRUE	TRUE	FALSE	56.10%
115	FALSE	FALSE	FALSE	TRUE	TRUE	FALSE	TRUE	47.60%
**116**	FALSE	FALSE	FALSE	TRUE	TRUE	FALSE	FALSE	**92.37%**
117	FALSE	FALSE	FALSE	TRUE	FALSE	TRUE	TRUE	28.35%
118	FALSE	FALSE	FALSE	TRUE	FALSE	TRUE	FALSE	40.95%
119	FALSE	FALSE	FALSE	TRUE	FALSE	FALSE	TRUE	56.10%
**120**	FALSE	FALSE	FALSE	TRUE	FALSE	FALSE	FALSE	**86.77%**
121	FALSE	FALSE	FALSE	FALSE	TRUE	TRUE	TRUE	27.00%
122	FALSE	FALSE	FALSE	FALSE	TRUE	TRUE	FALSE	56.10%
123	FALSE	FALSE	FALSE	FALSE	TRUE	FALSE	TRUE	44.17%
124	FALSE	FALSE	FALSE	FALSE	TRUE	FALSE	FALSE	82.18%
125	FALSE	FALSE	FALSE	FALSE	FALSE	TRUE	TRUE	26.31%
126	FALSE	FALSE	FALSE	FALSE	FALSE	TRUE	FALSE	64.71%
127	FALSE	FALSE	FALSE	FALSE	FALSE	FALSE	TRUE	60.87%
128	FALSE	FALSE	FALSE	FALSE	FALSE	FALSE	FALSE	79.07%

In the five highest-risk FFH accident scenarios, each variable marked as ‘True’ indicates the presence of the corresponding risk factor in that scenario, while ‘False’ indicates its absence. These combinations reveal critical patterns of risk co-occurrence that provide a practical basis for targeted safety interventions. For example, the most probable scenario (94.21%) involves the presence of D1 (Inadequate personal protection), E1 (Careless operation), and E2 (Low safety awareness of construction personnel), whereas A2 (Illegal and unauthorized construction), C1 (Non-standard scaffolding), C3 (Defective work equipment and facilities), and E3 (Physical or psychological problems of construction personnel) are absent. This indicates that even when conventional structural violations are not present, deficiencies in worker behavior and protective practices can independently generate high accident risk. The analysis of the remaining top scenarios reinforces this implication. The repeated appearance of E1 and E2 across the five highest-risk combinations highlights the critical role of human factors and the urgent need to improve on-site behavioral safety and awareness. Furthermore, the fact that high risk persists even in the absence of C1 (Non-standard scaffolding) suggests that organizational and behavioral weaknesses—rather than physical site conditions alone—can substantially elevate risk. These findings support the prioritization of data-informed, proactive safety measures focused on enhancing personnel training, compliance with personal protective equipment requirements, and behavioral oversight on construction sites.

## 5. Discussion

The objective of this study is to examine the probabilistic associations among risk variables and the corresponding risk scenarios of FFH accidents in construction projects. This investigation enhances our understanding of the mechanisms underlying accident risks and provides a scientific basis for developing prevention strategies.

Our findings indicate that, in descending order of influence, the primary risk variables are B2, E1, D1, E2, and A1. These results corroborate findings from Feng [12] and Zermane et al. [14], particularly regarding the dominance of organizational factors (B2, E1), while extending prior research by rigorously quantifying and ranking the impact magnitude of these risk variablesInadequate project safety supervision and enforcement (B2) emerged as the predominant influence, underscoring the critical role of negligence in safety supervision and inadequate safety implementation measures during the project construction process. Such oversight can lead to an escalation of risk factors across all project dimensions, culminating in FFH accidents that, in principle, could have been averted or otherwise should not have occurred. Careless operation (E1) is prevalent in construction work and may reflect inherent physiological factors [45]. Notably, our results suggest that everyday construction behaviors can also destabilize overall safety—a human factor largely unexplored in extant literature. Inadequate personal protection (D1) was identified as a critical risk factor in numerous prior investigations [14], and strategies such as computer vision-based monitoring have been introduced to address this issue [18]. Additionally, low safety awareness among construction personnel (E2) indicates that workers, often with limited educational backgrounds, may not fully recognize the hazards inherent in construction activities. Construction without proper construction qualification (A1) manifests in two forms. The first form is that the project review is not strict, which leads to such phenomena; the second form is that the project review is strict, but such personnel pass the review with the relevant qualification certificates of other people, or use fake relevant qualification certificates, which are difficult to identify.

The study further identified five high-risk scenarios that offer a comprehensive view of FFH accident risk. The first scenario posits that if D1, E1, E2 are true and A2, C3, E3, C1 are false, the risk probability of a FFH accident is 94.21%. This suggests that managers should avoid the simultaneous occurrence of Inadequate personal protection (D1), Careless operation (E1), and Low safety awareness of construction personnel (E2) during the construction process. Khan et al. [46] analyzed 2022 research papers and identified that the most common FFH hazards include a lack of protection, inadequate safety knowledge, loss of balance, and unsafe behaviors. Niu et al. [47] also noted that the more complex the interplay of risk factors, the greater the probability of adverse events. The findings of the present study are consistent with the documented occurrences of FFH accidents. For instance, the FFH accident report of the Beijing Miyun District Heating Service Center Office Building Renovation Project indicates that the workers’ failure to secure their safety belts using the designated safety ropes was attributed to a deficiency in safety awareness. This negligence resulted in the workers slipping and falling during the construction process, leading to the accident. The findings from these real-life scenarios substantiate the initial risk scenario postulated.

The probability of FFH accident risk in the second risk scenario is 92.37%, emphasizing Careless operation (E1) and Low safety awareness of construction personnel (E2), and the results are also consistent with the actual situation. For example, the FFH accident report of Guangdong Yu Times Electronics Co., Ltd. stated that the construction personnel had low safety awareness and climbed up the scaffolding of the outer wall after working on the inner wall and accidentally fell to the ground. This scenario is also in line with the findings of Mohammad Hanapi et al. [48] addressed the main issues of safety in construction work.

The results also indicate that the probability of the third risk scenario is 91.67%. This result provides implications for project managers to avoid the simultaneous occurrence of Inadequate personal protection (D1) and Careless operation (E1). Evidence for the third risk scenario is provided by the FFH accident report for the Huaqiao International Community Project in the Huaqiao Economic Development Zone, Kunshan, Jiangsu Province, which states that the construction worker fell through the gap under the outer guardrail while hanging the hook of his safety belt.

The risk probability of the fourth risk scenario is 86.77%. The fourth risk scenario emphasizes the operational behavior of the construction personnel (E1), and the result is consistent with the actual situation. For example, the FFH accident report of the First Outlet Middle Lot Residential Project in Kunshan Development Zone, Suzhou, Jiangsu Province, states that construction workers accidentally fell to the ground while climbing the vertical ladder.

The fifth risk scenario has a risk probability of 86.26%, in which Inadequate personal protection (D1) and Low safety awareness of construction personnel (E2) are emphasized, and the results are consistent with the actual situation. For example, the FFH accident report of Hangzhou, Jiangsu Province, Hangzheng Reserve No. 38 Residential Housing Project states that construction workers fell from a 6-meter scaffold while climbing the exterior wall scaffold without wearing safety belts. In summary, these findings elucidate the dynamic interplay among risk factors and scenarios in FFH accidents, thereby providing actionable insights for enhancing safety management practices in construction projects.

## 6. Limitations and future study

This study advances the understanding of FFH accident risk scenarios compared to existing literature; however, several limitations remain. First, the probabilities for risk variables and scenarios were derived from a dataset of 368 FFH accident reports. This relatively small sample size may not capture certain unique situations, thereby constraining the generalizability of the calculated risk probabilities. Second, while risk factors were extracted from these reports using Grounded Theory, the resulting descriptions may lack the level of detail necessary to fully represent the complexity of FFH risk scenarios. Third, the prediction model does not account for accident consequences, as the accident reports did not differentiate significantly in casualty levels. Moreover, relying on the frequency of documented risk variables could have led to the omission of scenarios that, although low in probability, might be associated with high hazards.Future research should aim to expand the sample size and include more detailed risk scenario variables. Such efforts would facilitate a more comprehensive exploration of accident consequences under varying risk scenarios. Additionally, developing risk probability databases for diverse scenarios could serve as a valuable resource, broadening perspectives and improving methodologies in the field of accident prevention.

Moreover, a key methodological limitation of Bayesian Networks lies in the conditional independence assumption, which, while improving interpretability and computational efficiency, may not fully capture all dependencies in real-world accident systems. Residual correlations may arise from latent or unmodeled factors, leading to biased probability estimates if relevant relationships are omitted. To mitigate this risk, the present study incorporated conditional independence testing during structure refinement and combined expert assessments with objectively reported accident investigation data to reduce subjective bias inherent in questionnaire-based judgments. Additionally, Dempster–Shafer evidence theory was applied to integrate multiple expert opinions, further enhancing the reliability of causal link identification. Nonetheless, we acknowledge that this assumption remains a trade-off inherent to BN modeling. Future research could explore hybrid approaches, such as integrating BNs with latent variable models or data-driven techniques (e.g., deep learning), to capture residual correlations while maintaining the interpretability of probabilistic graphical models.

Finally, while this study employed a manual Grounded Theory approach to extract risk factors from unstructured accident narratives, which enabled a rich and contextual understanding of FFH accidents, the process is inherently labor-intensive and time-consuming. This methodological choice facilitated in-depth engagement with the data and interpretive clarity, but may limit scalability and reproducibility in larger datasets. Recent studies have demonstrated the potential of Natural Language Processing (NLP) and Machine Learning (ML) techniques—such as BERT-based models, semantic clustering, and named entity recognition—for extracting causal and contextual features in a more systematic and scalable manner [48–50]. For example, Qi et al. [15] applied a BERT framework to construction safety narratives, highlighting its capacity to identify latent risk indicators with improved efficiency. Future research could benefit from incorporating these automated approaches—either as complementary tools to support the GT process or as standalone methods subject to validation—to improve coding consistency, expand dataset coverage, and enhance generalizability. Nevertheless, the interpretive depth offered by GT remains valuable, especially in complex, context-specific scenarios. A hybrid approach may thus offer a balanced pathway, provided that future models can accurately capture the nuanced interplay between situational context and risk emergence.

## 7. Conclusions

FFH accidents remain the most common type of accident in construction, and effective prevention hinges on accurately recognizing and identifying the associated risks. This study examined FFH accident risks and analyzed risk scenarios with varied probability levels. The primary conclusions are as follows:

(1) Using Grounded Theory, we identified five core dimensions that influence FFH accidents: illegal construction and permit deficiencies (A), inadequate safety management and supervision (B), site environment and equipment hazards (C), safety and personal protection deficiencies (D), and occupational safety risks for construction workers (E). These dimensions cover a wide range of factors from construction legality to personnel safety awareness, providing a comprehensive perspective on accident prevention. These dimensions span factors from construction legality to personnel safety awareness, providing a comprehensive framework for accident prevention.
(2) Under these dimensions, we identified 20 risk variables. The five most critical variables and their associated risk probabilities are as follows: the risk probability of ineffective enforcement of project safety supervision (B2) is 87.4%, the risk probability of careless operation (E1) is 61.5%, the risk probability of insufficient personal protection (D1) is 58.6%, the risk probability of poor safety awareness of construction personnel (E2) is 54.4%, and the risk probability of constructing the work without appropriate construction qualification (A1) is 51.4%. These findings offer a scientific basis for assessing and preventing FFH accidents.
(3) Five high-risk scenarios were identified. The first scenario, designated as Inadequate personal protection (D1), Careless operation (E1), Low safety awareness of construction personnel (E2) are True, Illegal and unauthorized construction (A2), Defective work equipment and facilities (C3), Physical or psychological problems of construction personnel (E3), Non-standard scaffolding (C1) are False, exhibits a 94.21% probability of an FFH accident. The second risk scenario, Careless operation (E1), Low safety awareness of construction personnel (E2) are True, Illegal and unauthorized construction (A2), Inadequate personal protection (D1), Defective work equipment and facilities (C3), Physical or psychological problems of construction personnel (E3), Non-standard scaffolding (C1) are False, has a 92.37% probability of an FFH accident occurring. The third risk scenario, Careless operation (E1), Inadequate personal protection (D1) are True, Illegal and unauthorized construction (A2), Defective work equipment and facilities (C3), Low safety awareness of construction personnel (E2), Physical or psychological problems of construction personnel (E3), Non-standard scaffolding (C1) are False, has a probability of 91.67% for an FFH accident. The fourth risk scenario, Careless operation (E1) is True, Illegal and unauthorized construction (A2), Inadequate personal protection (D1), Defective work equipment and facilities (C3), Low safety awareness of construction personnel (E2), Physical or psychological problems of construction personnel (E3), Non-standard scaffolding (C1) are False, and the probability of an FFH accident is 86.77%. The fifth risk scenario, Inadequate personal protection (D1), Low safety awareness of construction personnel (E2) are True, Illegal and unauthorized construction (A2), Defective work equipment and facilities (C3), Careless operation (E1), Physical or psychological problems of construction personnel (E3), Non-standard scaffolding (C1) are False, with a probability of FFH accident of 86.26%. These scenarios provide detailed insights into the dynamic interactions among risk variables and highlight specific combinations that lead to elevated accident probabilities.

This study advances theoretical understanding by identifying and quantifying the main risk variables associated with FFH accidents. Unlike prior research that has primarily focused on potential risk factors and conventional identification methods, our approach delves into the dynamics of risk scenarios. This expanded perspective provides new insights into the underlying mechanisms that drive FFH accidents, allowing for a more comprehensive framework for understanding accident causation.

On the practical side, the study offers valuable guidance for policymakers and project managers seeking to enhance construction safety. By quantifying the risk probabilities across different FFH accident scenarios, the findings support the development of targeted preventive measures. These risk scenario analyses not only inform more effective safety practices but also serve as a theoretical foundation for refining existing accident prevention techniques. Consequently, the study contributes both to the academic literature and to the practical toolkit available for improving safety in construction projects.

## Supporting information

S1 Text This file contains the anonymized dataset used in the study, including key variables extracted from the collected fall-from-height accident reports between 2014 and 2024. (DOCX)

S1 Consent Form This file provides the informed consent document used in the questionnaire survey, outlining participant rights and ethical considerations. (PDF)
