# Supplementary material for: Data-driven Bayesian networks for risk scenario mapping of Falls from height accidents
Source: PLoS One. 2025 Oct 14;20(10):e0334611. doi: 10.1371/journal.pone.0334611 (PMC12520409; doi:10.1371/journal.pone.0334611)
Supplement: S1 Consent Form — (PDF) [file pone.0334611.s002.pdf]

Survey on the Relationships among Influencing Factors of Falls from Height Accidents (FFHA)

Survey Description

Hello!

This is a questionnaire survey on the relationships among influencing factors of Falls from Height Accidents (FFHA) in construction. The purpose of this survey is to gain deeper insights into the causes and influencing factors of falls from height during construction work, so as to propose more effective preventive measures and ensure the safety of construction workers.

Your participation and valuable opinions will be of great help to our research. This questionnaire is anonymous and voluntary. All data collected will be used for research purposes only, and strict confidentiality will be maintained.

Thank you very much for taking the time to complete this questionnaire!

Start answering

建筑高空坠落事故（FFHA）各影响因素关系调查问卷

问卷说明

您好！

这是一份关于建筑高空坠落事故（FFHA）各影响因素关系的调查问卷。本问卷旨在深入了解建筑施工中高空坠落事故的成因及其影响因素，以便提出更有效的预防措施，保障施工人员的安全。

您的参与和宝贵意见将对我们的研究提供极大的帮助。本问卷采取不记名方式填写，所有数据仅用于科研目的，我们将严格保密。

感谢您在百忙之中抽空填写本问卷！

开始作答
